# Carrier Transport Properties of MoS_2_ Asymmetric Gas Sensor Under Charge Transfer-Based Barrier Modulation

**DOI:** 10.1186/s11671-018-2652-9

**Published:** 2018-09-04

**Authors:** Sun Jun Kim, Jae Young Park, SangHyuk Yoo, Palanivel Umadevi, Hyunpyo Lee, Jinsoo Cho, Keonwook Kang, Seong Chan Jun

**Affiliations:** 1 Department of Mechanical Engineering, Yonsei University, Seoul, 120-749 Republic of Korea; 2 Department of Computer Engineering, Gachon University, Gyeonggi-do, 461-701 Republic of Korea

**Keywords:** MoS_2_, Field-effect transistor, Schottky diode, Gas sensor, Contact effect, Schottky barrier

## Abstract

Over the past few years, two-dimensional materials have gained immense attention for next-generation electric sensing devices because of their unique properties. Here, we report the carrier transport properties of MoS_2_ Schottky diodes under ambient as well as gas exposure conditions. MoS_2_ field-effect transistors (FETs) were fabricated using Pt and Al electrodes. The work function of Pt is higher than that of MoS_2,_ while that of Al is lower than that of MoS_2_. The MoS_2_ device with Al contacts showed much higher current than that with Pt contacts because of its lower Schottky barrier height (SBH). The electrical characteristics and gas responses of the MoS_2_ Schottky diodes with Al and Pt contacts were measured electrically and were simulated by density functional theory calculations. The theoretically calculated SBH of the diode (under gas absorption) showed that NO_*x*_ molecules had strong interaction with the diode and induced a negative charge transfer. However, an opposite trend was observed in the case of NH_3_ molecules. We also investigated the effect of metal contacts on the gas sensing performance of MoS_2_ FETs both experimentally and theoretically.

**Electronic supplementary material:**

The online version of this article (10.1186/s11671-018-2652-9) contains supplementary material, which is available to authorized users.

## Background

In recent years, after the discovery of graphene, two-dimensional (2D) nanomaterials, which have vertically stacked layers connected by van der Waals (vdW) forces, have gained immense attention because of their unique properties [1–5]. Graphene, which is a layered hexagonal structure of carbon, with its unique properties such as high carrier mobility [6, 7], mechanical strength [8], and flexibility [9, 10], has opened up new avenues for nanoelectronic devices. Recently, transition metal dichalcogenides (TMDs), such as MoS_2_ and WSe_2_, have also been studied because of their higher band gaps as compared to that of graphene [11–15]. Monolayer MoS_2,_ with a thickness of 6.5 Å is the most widely known 2D-layered TMD. It shows a high mobility of up to ~ 200 cm^2^ V^−1^ s^−1^ [16] and on/off ratios exceeding ~ 10^8^ [17]. Furthermore, MoS_2_ is a semiconductor with an indirect band gap of 1.2 eV [18] in bulk and a direct band gap of 1.8 eV [19] in a single layer unlike graphene which has zero band gap. This zero band gap of graphene limits its application in nanoelectronic devices.

In order to develop MoS_2_ transistors with performance comparable to that of silicon-based devices, many limitations such as the quality of lattice state, fabrication, and contact resistance between the contact metal and MoS_2_ have to be overcome. Many of the previous studies in this context have focused on improving the electrical interaction at the interface of MoS_2_ and the metal electrodes. This is because contact-related properties include the potential difference, annealing conditions, and area. However, most of these studies assumed symmetric junctions and did not involve both experimental and theoretical analyses. In addition, it is difficult to analyze the carrier behavior of MoS_2_ under gas exposure conditions by only observing its band structure modulation. There is limitation for applying this simulation results because this basic band structure cannot provide any specific value for determining the modulation. Furthermore, although Schottky barrier height (SBH) is believed to be an important factor for determining the electrical response of MoS_2_ transistor under gas absorption, the previous studies did not analyze the effect of SBH both theoretically and experimentally.

In this study, we fabricated MoS_2_ FETs with asymmetric electrodes, Al and Pt, to observe carrier transport through the Schottky barrier under gas exposure conditions. First, the work function difference in the devices was geometrically mapped by measuring their surface potentials using Kelvin probe force microscopy (KPFM). To design the MoS_2_ Schottky diode, the contact effect of the MoS_2_/metal interface was analyzed under ambient conditions both theoretically (density functional theory (DFT) calculations) and experimentally (electrical measurements of the symmetric and asymmetric MoS_2_ FETs). The electrical response of the diode was measured under gas exposure conditions. This electrical response was then compared with the theoretically calculated SBH change values which makes possible to understand the modulation numerically. The findings of this study provide an insight into the interaction of gas molecules and the MoS_2_/metal contact interface in MoS_2_-based gas sensing devices.

## Method

### Fabrication of MoS_2_ Devices

We fabricated the MoS_2_ Schottky devices using a facile mechanical transfer method. Few-layered flakes of MoS_2_ were exfoliated from its bulk crystal, which was purchased from SPI supplies. Using polydimethylsiloxane (PDMS) (“Sylgard 184”, Dow corning), MoS_2_ was transferred to highly doped Si/SiO_2_ substrates. Pt and Al electrodes (100 nm thick) were deposited on the sample films and were patterned by electron beam lithography using a field emission scanning electron microscope (FE-SEM) (JSM-7001F, JEOL Ltd.). The performance of the MoS_2_ devices was evaluated by measuring their source/drain and source/gate voltage modulations (Keithley 2400 source meter) at room temperature.

### Surface Potential Measurement

The surface potential of the devices was measured by the interleave mode of electric force microscopy (Nanoscope IV, Veeco) using a PtIr-coated silicon probe tip (SCM-PIT, Veeco) at ambient air condition of 25 °C and 1 bar. The first scan of the tip examined the surface topology of the devices. A subsequent second scan was carried out to measure the electrostatic force between the device surface and the tip.

### DFT Calculations

A $\sqrt{3}\times \sqrt{3}$ supercell of MoS_2_ was prepared with three Mo atoms and six S atoms (Fig. 3a). A vacuum spacing of 15 Å was defined in order to prevent the interaction of the images. The lattice constant was calculated to be 3.184 Å, which is in good agreement with the experimental value (3.160 Å). Substrates with six layers of Al or Pt metal atoms (with (111) free surface) were fabricated to construct the interface between the metals and monolayer MoS_2_. The lattice constants of Al and Pt substrates were computed to be 4.070 and 3.973 Å, respectively. After the geometry optimization of each structure, monolayer MoS_2_ was deposited on the substrate and the configuration was optimized again. A lattice mismatch between MoS_2_ and the metal substrates was observed because the monolayer of MoS_2_ stretched during the geometry optimizations. The structure of monolayer MoS_2_ with gas molecules (including NO_2_ and NH_3_) was also constructed and optimized using a $\sqrt{3}\times \sqrt{3}$ supercell.

DFT calculations were performed by using VASP (Vienna ab initio simulation package) [20–23]. GGA (generalized gradient approximation)–PBE (Perdew-Burke-Ernzerhof) to exchange-correction functional of PAW (Projector Augmented-wave) method was used with vdW corrections [24–27]. The cutoff energy for the basis set was extended to 500 eV for all the calculations. For the self-consistency and band structure calculations, the electronic energy convergence and atomic force criteria were set to 10^−5^ eV and 0.02 eV/Å, respectively. The K-points for Brillouin-zone sampling were 8 × 8 × 1 (with Gamma (Γ) point centered). For measuring the vdW interactions between the gas molecules and MoS_2_, the DFT-D2 method of Grimme was used [28].

## Result and Discussion

**Fig. 1:**
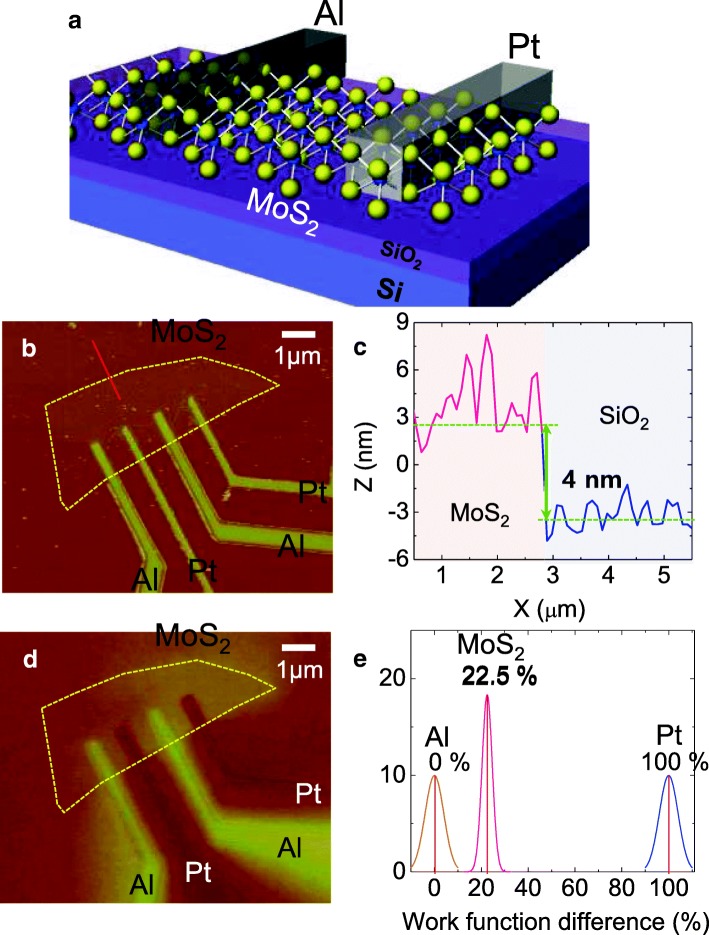
**a** Schematic diagram of the MoS_2_ Schottky diodes with Al and Pt contacts. **b** AFM image of the MoS_2_ Schottky diode device with asymmetric metal electrodes (Al/Pt). **c** Cross-sectional analysis of the device for measuring the thickness of MoS_2_ layer. **d** Surface potential image of the same device. **e** Normalized distribution of the relative surface potentials of MoS_2_, Al, and Pt

We prepared MoS_2_ devices with two types of electrodes (Al and Pt) and characterized their morphology and thickness using atomic force microscopy (AFM) (Fig. 1a). Figure 1b shows the height of the MoS_2_ layer along the cross-section line (shown by the red line in Fig. 1a). The thickness of the MoS_2_ sample was 4 nm. To demonstrate the work function difference in the MoS_2_ devices with symmetric and asymmetric electrodes, we employed KPFM to measure the contact potential difference between MoS_2_ and the probe tip. When the probe tip and sample were close enough, an electrostatic force was applied because of the work function difference between them. The relation between the electrostatic force and work function of the two materials is as follows:

$$ {F}_{\mathrm{electrostatic}}=\frac{q_{\mathrm{s}}{q}_{\mathrm{t}}}{4{\pi \varepsilon}_0{z}^2}+\frac{1}{2}\frac{dC}{dz}{\left({V}_{\mathrm{applied}}-{V}_{\mathrm{contact}}\right)}^2 $$

where *dC*/*dz* is the derivative capacitance between the sample and the tip, *q*_s_ is the surface charge, and *q*_t_ is the charge of the tip. *V*_contact_ can be characterized by the surface potential value [29]. Using the surface potential value, we calculated the work function as

$$ {V}_{\mathrm{contact}}={\Phi}_m-{\chi}_s-\varDelta {E}_{fm}-\varDelta \Phi $$

where Φ_*m*_ is the work function of the probe tip, *χ*_*s*_ is the electron affinity, *ΔE*_*fn*_ is the Fermi-level position from the lowest level of the conduction band, and *Δ*Φ is the modified band bending.

The surface potential mapping of the devices is shown in Fig. 1c. We added the work function value (4.85 eV) of PtIr-coated Si tip to get the work function of electrode and channel part [30]. Then, normalization process was followed by positioning the percentage value of MoS_2_ between Pt and Al as shown in Fig. 1d. The difference between the surface potentials of Al and MoS_2_ was 22.5%, which is smaller than that between the surface potentials of Pt and MoS_2_ (100%). Unlike Pt, Al has a work function comparable to that of MoS_2_. This is because the surface potential of Al is comparable to that of MoS_2_. Since, MoS_2_ and Al have similar work functions, they can form Ohmic contacts. MoS_2_ and Pt exhibit Schottky contacts because of their large surface potentials. Further studies should be followed to confirm whether the potential modulation occurs under gas absorption for understanding gas sensing mechanism.

**Fig. 2:**
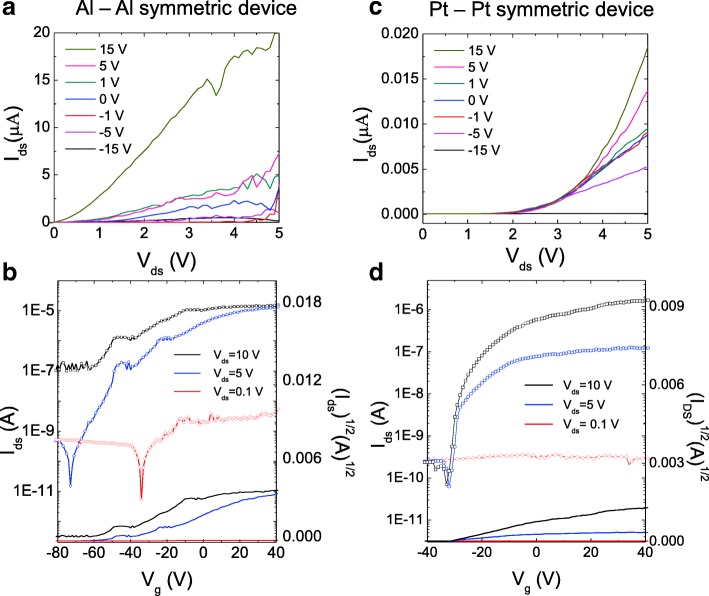
**a** Output curve and **b** transfer curve of the MoS_2_ device with Al-Al symmetric electrodes. **c** Output curve and **d** transfer curve of the same device with Pt-Pt symmetric electrodes

To compare the asymmetric junction characteristics of the devices, the current–voltage characteristics of the devices with Al and Pt contacts over the gate voltage range of − 15–15 V are shown in Fig. 2a, c, respectively. The MoS_2_ device with Al contact showed a linear drain current which was much higher than that of the device with Pt contact. The current of the Al contact was more than 1000 times higher than that of the Pt contact. This suggests that the SBH of devices with low-work function metal contacts is low. To further investigate the effect of metal contacts on the MoS_2_/metal interface of the devices, their transfer characteristics at different forward bias voltages (0.1, 5, and 10 V) were measured (Fig. 2b, d). In both the cases (Al and Pt contacts), the transfer curves of MoS_2_ showed the characteristics of n-type semiconductors, i.e., the current level at positive gate voltages was higher than that at negative gate voltages [31]. At the source-drain bias of 0.1 V, only the device with Al contact showed the on-off tendency. When the bias was increased to 5 V, the on-off ratios of the Al and Pt contacts were approximately 10^6^ and 10^3^, respectively. As the bias voltage approached 10 V, the off function of the device with Al contact became disabled, while the on-off ratio of the Pt contact increased. This suggests that in order to achieve gas sensing devices with the desired performance over a specific current range, it is imperative to use appropriate metal contacts. In order to determine the threshold voltage of the devices, the $\sqrt{I_{DS}}$ versus gate voltage curve was added to their transfer curves (Fig. 2b, d). This is because it is easier to measure the threshold voltage by smoothing the fluctuations of the $\sqrt{I_{DS}}-{V}_g$ line. The threshold voltage induced by the $\sqrt{I_{DS}}-{V}_g$ line for the device with Al electrode was about − 70 V, while that for the device with Pt electrode was about − 30 V (Fig. 2a, c). The threshold voltage of the device with Al contact was much lower than that of the device with Pt contact. This can be attributed to the lower Schottky height of the Al/MoS_2_ interface as compared to that of the Pt/MoS_2_ interface. In addition, the threshold voltage of the device with Al contact was strongly modulated by the source-drain voltage. On the other hand, no significant change was observed in the threshold voltage of the device with Pt contact with the drain-source voltage.

**Fig. 3:**
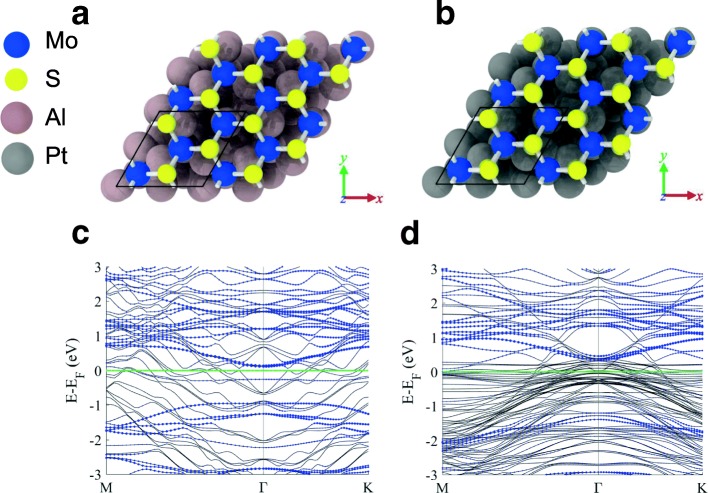
**a**, **b** The 3D models of MoS_2_ on Al and Pt substrates, which were used in DFT calculations. **c**, **d** The band structures of these models. Green lines indicate the Fermi energy set by taking zero as the work function of the vacuum level. Blue dashes correspond to the energy bands of monolayer MoS_2_. Difference between the value of green lines and the minimum value of blue dashes on the conduction band site is SBH [38]

To theoretically analyze the electrical states at the metal/MoS_2_ interface, DFT calculations were carried out using a MoS_2_-on-Al configuration (Fig. 3a, b). Table 1 lists the lattice mismatches and distance *h* between MoS_2_ and the metal substrates. The values obtained in this study were consistent with those reported previously [32]. The band structures of MoS_2_ with the Al and Pt substrates are shown in Fig. 3c, d, respectively. Work function and SBH values are summarized in Table 1. Work function and SBH values are summarized in Table 1. Work function of MoS_2_ with Pt substrate (5.755 eV) is well-matched previous results (5.265 eV) [32]. The value of SBH for the device with Al substrate was 72% lower than that for the device with Pt substrate. The reason of SBH difference results from work function difference between Al and Pt; work function of Al is 64% lower than that of Pt. [33] Thus, Al/Pt asymmetric contact systems can function as diodes.

**Table 1:** Lattice mismatch was calculated using the lattice constant of MoS_2_ (*a* = 5.514 Å). Distance *h* is the difference between the averaged *z*-position values of S and Al at the interface. Work function was computed by the equation, *W* = *E*_fermi_ − ϕ_vac_, *E*_fermi_ is the Fermi energy of the total system (MoS_2_ + metal substrate), and *ϕ*_vac_ is the vacuum potential of the system. SBH was calculated as mentioned in the legend of Fig. 3

	Metal substrate	Al (111)	Pt (111)
Structural parameters	Lattice mismatch (%)	4.38	1.39
	Distance *h* (Å)	2.517	2.218
Electronic parameters	Work function (eV)	4.680	5.265
	SBH (eV)	0.1423	0.506

**Fig. 4:**
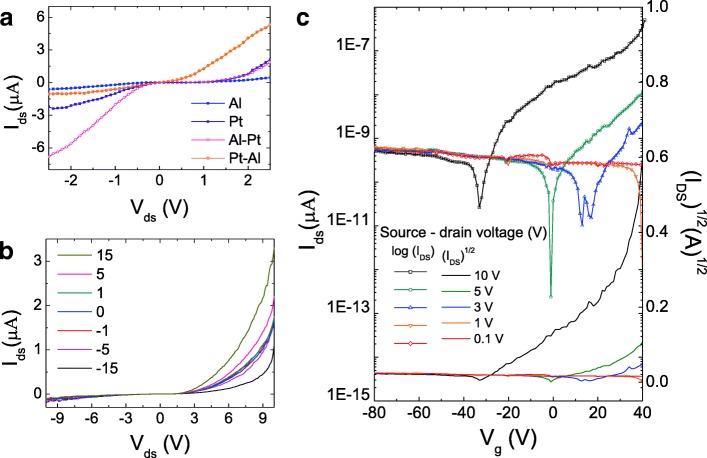
**a** I-V_DS_ curve of the MoS_2_ device with symmetric electrodes (Al-Al, Pt-Pt) and asymmetric electrodes (Al-Pt). **b** Transfer curve and **c** output curve of the asymmetric devices

To further examine the performance of Al/Pt asymmetric systems, we fabricated Al/Pt asymmetric metal electrodes on MoS_2_ Schottky devices. Figure 4a shows the current–voltage characteristics of the MoS_2_ devices with Al-Al, Pt-Pt, Al-Pt, and Pt-Al contacts (as the order of source and drain). Unlike the symmetric curve of the Al-Al and Pt-Pt devices, the asymmetric diode showed rectifying characteristics in the direction of the MoS_2_/Al contact. To investigate the effect of charge transfer on the performance of the devices, we observed their drain currents as a function of the gate bias (Fig. 4b). The transfer curves corresponding to the source-drain voltage were also obtained (Fig. 4c). Figure 4c shows that the threshold voltage shifted from 40 to − 40 V with an increase in the source-drain voltage. A similar trend was observed in the case of the symmetrically Al-contacted device. This implies that the Al/MoS_2_ contact side affected the carrier transport of the device more than the Pt/MoS_2_ contact side.

**Fig. 5:**
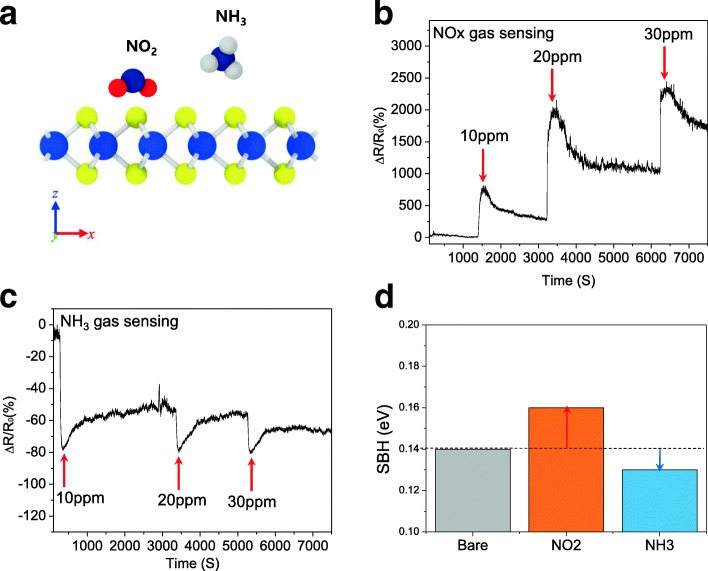
**a** Schematic diagram of MoS_2_ and the gas molecules, which were used for simulation. **b**, **c** The resistance changes of the MoS_2_ Schottky diode upon NO_*x*_ and NH_3_ exposure, respectively. **d** Theoretically calculated SBH of the MoS_2_/metal interface under ambient and gas exposure conditions (NO, NO_2_, and NH_3_)

The real-time gas response of the MoS_2_ Schottky diode was measured to observe its Schottky barrier modulation with charge transfer. The gas sensitivity of the diode was calculated using the following equation:

$$ \frac{\Delta R}{R_{\mathrm{air}}}=\frac{R_{\mathrm{gas}}-{R}_{\mathrm{air}}}{R_{\mathrm{air}}} $$

where *R*_air_ and *R*_gas_ represent the resistance of the MoS_2_ Schottky diode under ambient and gas exposure conditions, respectively. Figure 5 shows the gas sensing ability (change in resistance with time) of the MoS_2_ Schottky device for NO_*x*_ and NH_3_ molecules (10, 20, and 30 ppm) at an applied source-drain bias of 3 V. Since NO_*x*_ is a strong electron acceptor, and hence is a p-doping material, the resistance of the device increased with an increase in the gas exposure because of the negative charge injection at the interface of MoS_2_ [34]. The p-doping of MoS_2_ increased its Schottky barrier, which in turn increased the contact resistance at the MoS_2_/metal interfaces. The gas absorption dependence of the signal response was also observed. The sensitivity of the device increased with an increase in the gas concentration, indicating an increase in its charge transfer. The resistance of the device on the other hand decreased upon exposure to NH_3_ (Fig. 5c). This is because NH_3_ donates electrons to MoS_2_, thus decreasing its Schottky barrier [35]. The measured gas sensitivity of NH_3_ was much lower than that of NO_*x*_, indicating that charge transfer in the presence of NH_3_ was lower than that in the presence of NO_*x*_ [36]. In addition, a slight dependence of gas concentration was also observed after the current fluctuation in each step. With an increase in the NH_3_ concentration, the resistance of the device decreased. This is because the MoS_2_/Al interface showed lower SBH values at higher NH_3_ concentrations. To confirm these results theoretically, we calculated the SBH of the MoS_2_/Al interface, which was in contact with various kinds of gas molecules (Fig. 5d). Kang et al. previously discussed about Schottky barrier theory of MoS_2_/metal contact and explained the carrier transport through contact side by using three types of model [37]. According to band diagram illustrated in this paper, Schottky barrier modulation occurs at the boundary of electrode and channel. So, we designed the composite structure which has uniformly distributed Schottky barrier to facilitate the observation of Schottky barrier modulation according to gas absorption. However, the model is not applied to all situations. Type 3 showed that Schottky barrier was not formed at the directly contacted interface of MoS_2_ and metal because of the strong metallization effect. The metals which have strong adhesion with MoS_2_ like Ti and Mo are classified as Type 3. To explore various contact effects in the metal/MoS_2_ composite, careful consideration should be followed to design the model structure (Additional file 1: Figure S1 and S2). Only the Al side was selected for calculating the barrier height because the barrier with Pt electrode did not disturb the carrier transport under the forward bias. NO_2_ and NH_3_ were selected for the modulation of the Schottky barrier of the MoS_2_/Al interface. This Schottky barrier was compared with that observed in pristine condition (Table 1). The theoretically calculated barrier heights for NO_2_ and NH_3_ were 0.16 and 0.13 eV, respectively. This result shows that NO_2_ and NH_3_ induced charge transfer in different directions. The Schottky barrier was affected more by NO_2_ than by NH_3_. These results were consistent with the experimental results. The results also demonstrate that MoS_2_ Schottky diodes have great potential to be used in next-generation gas sensing devices.

## Conclusion

In this study, we investigated the effect of the contact material on the properties of MoS_2_ asymmetric FETs under ambient and gas exposure conditions. The KPFM results showed that Pt had the highest work function followed by MoS_2_ and Al. The DFT results predicted that the SBH of the MoS_2_/metal interface was higher for the metal with higher work function. This is consistent with the experimental results obtained for the symmetric (Al-Al and Pt-Pt) and asymmetric (Al-Pt) FETs fabricated in this study. The absorption of NO_*x*_ resulted in a strong gas response and in an increase in the resistivity of the device. Opposite trends were observed in the case of NH_3_. These results were consistent with the theoretically calculated SBH values. This study emphasizes on the importance of choosing appropriate metal contacts for developing MoS_2_ gas sensors with desired performance.

## Additional file

Additional file 1: **Figure S1.** Configurations of adsorption molecules on the MoS_2_ with Al substrate. **Figure S2.** Band structure calculation of pristine MoS_2_ with gas adsorption. (DOCX 310 kb)

## Abbreviations

AFM: Atomic force microscopy
DFT: Density functional theory
FET: Field-effect transistor
KPFM: Kelvin probe force microscopy
SBH: Schottky barrier height
TMDs: Transition metal dichalcogenides
*V*_ds_: Source-drain voltage
vdW: van der Waals
