# Supplementary material for: Carrier Transport Properties of MoS2 Asymmetric Gas Sensor Under Charge Transfer-Based Barrier Modulation
Source: Nanoscale Res Lett. 2018 Sep 4;13:265. doi: 10.1186/s11671-018-2652-9 (PMC6123339; doi:10.1186/s11671-018-2652-9)
Supplement: Supplementary file 1 — Figure S1. Configurations of adsorption molecules on the MoS2 with Al substrate. Figure S2. Band structure calculation of pristine MoS2 with gas adsorption. (DOCX 310 kb) [file 11671_2018_2652_MOESM1_ESM.docx]

Carrier transport properties of MoS_2_ asymmetric gas sensor under charge transfer-based barrier modulation

Sun Jun Kim^a,‡^, Jae Young Park^a,‡^, SangHyuk Yoo^a,‡^, Palanivel Umadevi, Hyunpyo Lee^a^, Jinsoo Cho ^b,^*, Keonwook Kang^a,^*, and Seong Chan Jun^a,^**

^a^ Department of Mechanical Engineering, Yonsei University, Seoul 120-749, Republic of Korea

^b^ Department of Computer Engineering, Gachon University, Gyeonggi-do 461-701, Korea

* Corresponding author

** Corresponding author

^‡^These authors contributed equally to this article.

**Email address**

kimarcel@daum.net (Sun Jun Kim), qkrwodudno1@naver.com (Jae Young Park), moonbear88@gmail.com (SangHyuk Yoo), renumaphy@gmail.com (Palanivel Umandevi), hpleei12@naver.com (Hyunpyo Lee), jscho@gachon.ac.kr (Jinsoo Cho), kwkang75@yonsei.ac.kr (Keonwook Kang), Seong Chan Jun ([scj@yonsei.ac.kr](mailto:scj@yonsei.ac.kr))

**Configurations of adsorption molecules on the MoS_2_ with Al substrate**

Initial structures are constructed with reference to previous DFT results [1, 2]. Final configurations of NO, NO_2_ and NH_3_ on the MoS_2_ with Al substrate are shown in Fig S1 (a)~(c), respectively. Band structures and Schottky Barrier Height(SBH) are computed by using Figure S1.


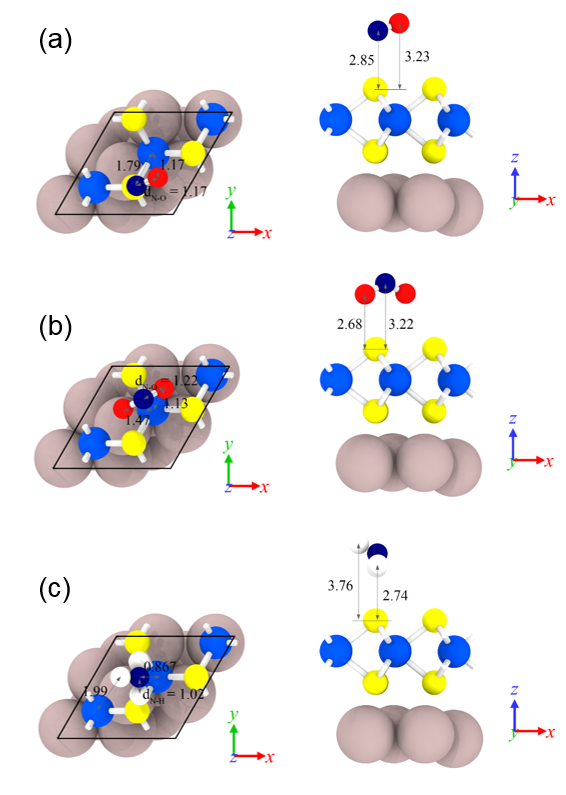


Figure S1. (a) adsorbate NO (b) adsorbate NO_2_ (c) adsorbate NH_3_: Final configurations of adsorbates on MoS2 with Al substrate. Units are Å. d_N-O_ and d_N-H_ means that distance between two atoms in 3D. Other numbers describe distance between atoms linked by arrow in xy or zx plane.

**Band structure calculation of prisitine MoS_2_ with gas adsorption**

Figure S2.(a) is band structure of pristine MoS_2_. Figure S2.(b) is the band structure MoS_2_-NH_3_. We can find that there is no significant change. On the contrary, especially NO adsorption, fermi level is upshifted considerably by the NO_x_ gases in Figure S2.(c) and (d). Increasing the fermi level results in effect of the n-type doping. In previous research, when both surfaces of MoS_2_ is exposed to NO_x_ gases, there will be charge transfer from MoS_2_ to NO_x_ gases, which indicates NO_x_ works as an accepter. On the other hand, If NH_3_ gases are around the surfaces of MoS_2_, it was reported that no meaningful changes on the band structure near the fermi level did not occurred and in DFT calculation, but works as the electron doner [1–2]. From the DFT calculation results and previous studies, we can expect that NH_3_ gases can explain the decrement of total resistance during gas sensing experiments by the drop of intrinsic and contact resistance.

However, although we observed some meaningful change in the gas absorption, there was no specific value for explaining this change. That’s why Schottky barrier analysis is worthy for understanding device characteristics numerically.


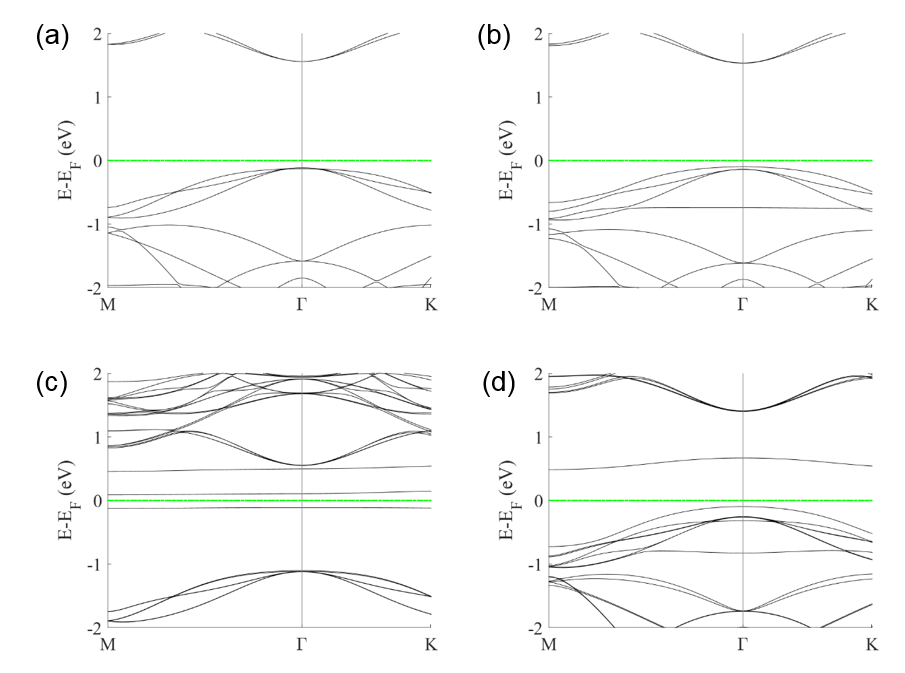


Figure S2. Band structure (a) pristine MoS_2_ (b) MoS_2_-NH_3_ (c) MoS_2_-NO (d) MoS_2_-NO_2_

**Reference**

[1] S. Zhao, J. Xue, W. Kang (2014) Gas adsorption on MoS2 monolayer from first-principles calculations. Chem. Phys. Lett., 595-596;5-42.

[2] Q. Yue, Z. Shao, S. Chang, J. Li (2013) Adsorption of gas molecules on monolayer MoS2 and effect of applied electric field. Nanoscale Res Lett 8;425.
